# Creep Deformation by Dislocation Movement in Waspaloy

**DOI:** 10.3390/ma10010061

**Published:** 2017-01-12

**Authors:** Mark Whittaker, Will Harrison, Christopher Deen, Cathie Rae, Steve Williams

**Affiliations:** 1 Institute of Materials, Bay Campus, Swansea University, Swansea SA1 8EN, UK; w.harrison@swansea.ac.uk (W.H.); 481652@swansea.ac.uk (C.D.); 2 Department of Materials Science and Metallurgy, University of Cambridge, Cambridge CB3 0FS, UK; cr18@cam.ac.uk; 3 Rolls-Royce plc, Elton Road, Derby DE24 8BJ, UK; steve.williams@rolls-royce.com

**Keywords:** creep, dislocations, Wilshire equations, forest hardening

## Abstract

Creep tests of the polycrystalline nickel alloy Waspaloy have been conducted at Swansea University, for varying stress conditions at 700 °C. Investigation through use of Transmission Electron Microscopy at Cambridge University has examined the dislocation networks formed under these conditions, with particular attention paid to comparing tests performed above and below the yield stress. This paper highlights how the dislocation structures vary throughout creep and proposes a dislocation mechanism theory for creep in Waspaloy. Activation energies are calculated through approaches developed in the use of the recently formulated Wilshire Equations, and are found to differ above and below the yield stress. Low activation energies are found to be related to dislocation interaction with γ′ precipitates below the yield stress. However, significantly increased dislocation densities at stresses above yield cause an increase in the activation energy values as forest hardening becomes the primary mechanism controlling dislocation movement. It is proposed that the activation energy change is related to the stress increment provided by work hardening, as can be observed from Ti, Ni and steel results.

## 1. Introduction

The prediction of long-term creep properties based on short timescale experiments is rated as the most important challenge to the UK Energy Sector in the recent UK Energy Materials Review [1]. However, despite extensive research performed over more than half a century, no single approach linking micro-mechanical behaviour to macroscopic properties and material behaviour has gained widespread support [2,3,4,5,6,7,8,9]. It is clearly desirable that the framework of any successful theory derive from, and be consistent with, microstructural observations. Furthermore, any successful theory should be able to predict a range of properties, such as time to fracture, minimum creep rate, applied stress and creep strain evolution. In many approaches, the importance of creep strain evolution is overlooked since only total service life (normally time to fracture) is the critical parameter. Typically, static components, such as steam generation and transport plant found in the power generation sector, fall into this category, however, other applications of rotating components require more detailed information on the evolution of creep strain as deformation can lead to contact and therefore material damage. In gas turbines, rotating components operating in a high temperature gas stream have blade clearances minimised in order to promote higher efficiency. Creep deformation of both discs or blades will lead to contact with casings and promote wear fatigue and/or tip cracking.

Traditional methods of creep lifing have been based on power law type equations, in which the minimum creep rate ($\dot{\varepsilon }$_m_) and the rupture time (t_f_) vary with stress (σ) and temperature (T) according to the relationship

(1) $$M/t_{f}={\dot{\varepsilon }}_{m}=A\cdot \sigma^{n}\cdot \exp(‒Q_{c}/\mathrm{RT})$$

where Q_c_ is the activation energy for creep, R is the gas constant and M is the Monkman Grant constant. The description of the equation in terms of minimum creep rate, rather than secondary creep rate is important, since recent research [10,11,12] has questioned the existence of a true steady-state (secondary) creep rate, as the decaying primary stage is immediately offset by the accelerating tertiary phase, resulting only in a minimum, and not a steady state, creep rate. This smooth transition from primary to tertiary creep is consistent with models proposed by Evans [13], Dyson [14] and Wu [15].

In Equation (1) [16], the parameters (A and M), the stress exponent (*n*) and the activation energy for creep (Q_c_) vary depending on the stress and temperature imposed, despite being initially proposed as constants. The dominant creep mechanism is identified by comparing experimentally measured and theoretically predicted values of *n* and Q_c_. With pure metals, a decrease from *n* ≅ 4 to *n* ≅ 1 with decreasing stress has been widely attributed to a change from diffusion-controlled dislocation processes to diffusional creep mechanisms not involving dislocation movement. Clearly, the variation in *n* and Q_c_ values offers significant difficulties in producing a comprehensive lifing methodology capable of holistic predictions over a range of temperatures and stresses, but this approach has also encountered a number of more specific difficulties. Firstly, the contribution of non-dislocation based diffusional creep mechanisms has recently been questioned [17]; Secondly, at high stresses with alloys strengthened by dispersions of fine precipitates or insoluble particles, *n* can take values substantially in excess of 4 and the value for Q_c_ significantly exceeds that of the activation energy for lattice diffusion, Q_SD_. No generally accepted physical interpretation of these anomalously large *n* and Q_c_ values has been agreed [5].

Several modern approaches to creep lifing normalise the stress by the Ultimate Tensile Strength (UTS) [14,15], and a recent approach that has been particularly successful is the use of the Wilshire equations [16,17,18,19,20]. The Wilshire equations acknowledge that t_f_ → 0 as σ → σ_TS_, and t_f_ → ∞ as σ → 0; as a result, the equations produce more appropriate curve shapes. Temperature is accounted for by use of an activation energy, Q_c_*, that takes account of the variability embodied in the UTS and is thus slightly lower than a conventional activation energy fitted to the same data.

Indeed, approaches to creep lifing utilising the Wilshire equations have been extremely successful [18,19,20]. Initial investigations involving aluminium alloys and pure copper have more recently been supplemented by predictions of long-term creep behaviour in key power generation steels as P22/T22, P23/T23 [21], P91, P92, Grade 122 [22], HK40, HP40 [23] and Type 316 stainless steel [24].

Significant features have consistently arisen in fitting the constants for the Wilshire equations to the data sets of these alloys. Datasets can be fitted more accurately to two distinct sets of parameters in terms of minimum creep rate or rupture time with the ‘break points’ being a function of stress. A break point is commonly found to occur at stresses approximately equal to the yield stress of the material, and this is assumed to be related to a change in the dislocation behaviour at this point.

More recently, application of the Wilshire Equations to engineering materials has indicated that not only should the material fitting constants k_1_ and u vary either side of a break point, approximately equal to the yield stress, but that the apparent activation energy (Q_c_*) should also differ. A wide range of materials [24,25,26,27] have now been studied for which optimised fits are obtained when the value of the apparent activation energy (Q_c_*) is allowed to vary either side of the break point. Interestingly, the most significant changes in the apparent activation energy observed have been in stainless steel grade 316, with values of 250 kJ/mol observed above the yield stress, decreasing to 150 kJ/mol below. It is useful to compare these values with Titanium-6-4, where no change in activation energy was observed, and Udimet 720 where only a small change (40 kJ/mol) was observed. When considering however, that 316 stainless steel shows an extremely high rate of strain hardening under monotonic loading, and Ti-6-4 shows extremely low rates of strain hardening, it becomes clear that dislocation interactions must play a significant role in the determination of this value, and hence the behaviour of the material. This paper examines the deformation structures observed above and below the threshold in order to rationalise this apparent change in behaviour.

## 2. Experimental Methods

The material selected for the programme was Waspaloy. It is a precipitation hardened alloy which was developed from the Nimonic series of alloys. It has considerable strength and corrosion resistance at temperatures up to 870 °C above which intergranular oxidation compromises performance. The nominal chemical composition of Waspaloy is shown in Table 1.

The material selected for this programme had undergone a forging heat treatment, which would typically involve 995 to 1040 °C (sub-solvus) for 4 h, air cooled, stabilisation at 845 °C for 4 h, air cooled and aging at 760 °C for 16 h, air cooled [29].

Creep testing during the programme was completed in air on a Mayes constant stress creep machine with a loading lever ratio of 15:1 for stresses above 10 MPa (discounting the onset of tertiary failure and necking). Specimen strains were monitored using extensometers incorporating a pair of differential capacitance transducers capable of resolving changes in gauge length to 10 nm.

Testing was carried out at temperatures ranging from 550 to 800 °C (823–1073 K) measured using 2 R-type thermocouples, to an accuracy of ±3 °C between thermocouples. All loading, temperature and strain monitoring systems are calibrated annually, in line with BS EN ISO 7500-2 [30] and BS EN ISO 9513 [31].

**Figure 1:**
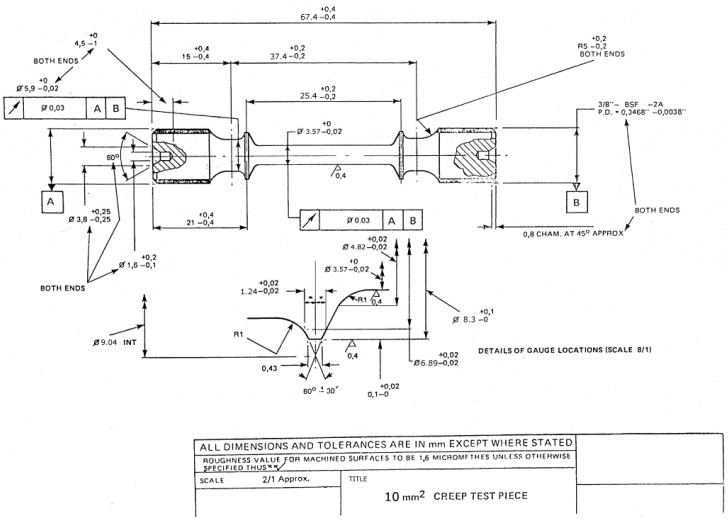
Creep specimen design.

For one set of experiments, tests were stopped at the minimum strain rate condition, frequently recalculated during test progression using the secant method, then air cooled under reduced load. For a second set of experiments, tests were stopped at 1% creep strain accumulation and furnace cooled with the load removed. For minimum strain rate experiments, test specimens were machined down to 3 mm gauge diameter from the standard specimen design shown in Figure 1, to avoid excess machining post testing. No effect of environment would be expected from the different gauge diameters, due to the short timescales of these tests, at only intermediate temperatures.

For 1% strain experiments, test specimens utilised a standard specimen shown in Figure 1. These discs were then spark eroded to produce a 3 mm diameter disc. In preparation for TEM analysis, thin discs were cut from within the gauge length of the crept specimen, to an approximate thickness of 500 microns to 1 mm, using a high precision cutting wheel. These discs were then mounted using MWH135 mounting wax and ground to a thickness of 150–200 microns. The thin discs were subjected to twin-jet electropolishing using a solution of 10 vol % perchloric acid in methanol at −5 °C and 20.5 V.

## 3. Results

**Figure 2:**
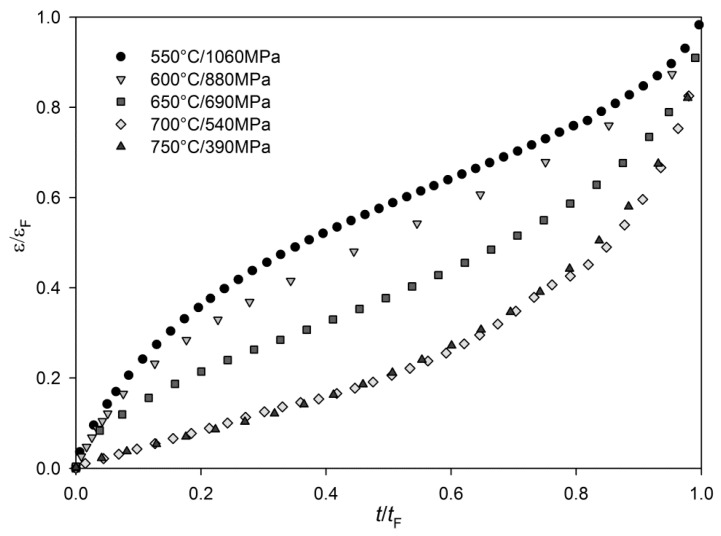
Creep curves may take significantly different shapes dependent on applied stress and temperature.

High precision extensometry allowed for the recording of creep curves in the experiments undertaken and it is clear from Figure 2 that the creep curve shape for tests showing similar rupture times can vary significantly with temperature and stress. Curves generated at lower temperatures such as 550 °C generally are undertaken at stresses in excess of the yield stress of the material to produce appropriate rupture times, and therefore a significant amount of primary creep strain is evident as dislocations are continually generated under this high stress. At higher temperatures and lower stresses, creep deformation occurs more readily under stresses below the yield stress, and deformation is accommodated by the movement of pre-existing dislocations subject to processes such as climb and cross slip, with most deformation accommodated in grain boundary zones. This results in a reduced primary stage, but extended tertiary creep. The difference in the shape of the curves is significant, as shown in Figure 2, and illustrates the requirement for prediction of the full creep curve where possible, so that creep strain is well defined under all conditions. With appropriate models in place, applications where time to a predefined strain is more critical than total rupture time can be suitably accommodated.

**Figure 3:**
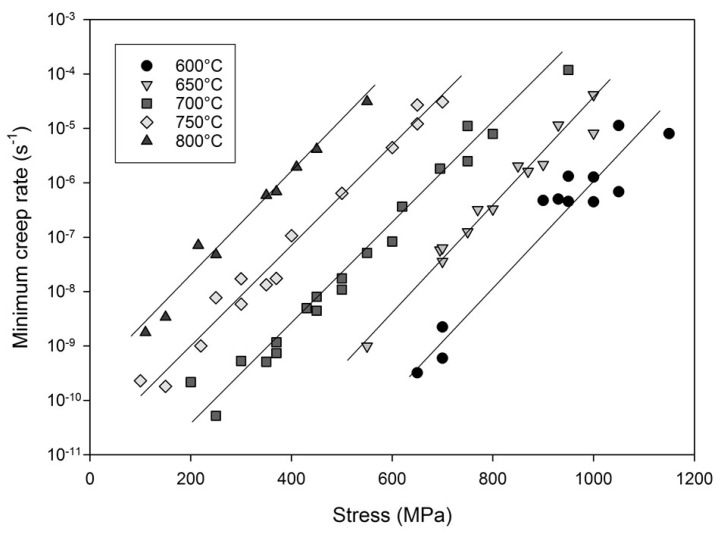
The relationship between stress and minimum strain rate in Waspaloy.

**Figure 4:**
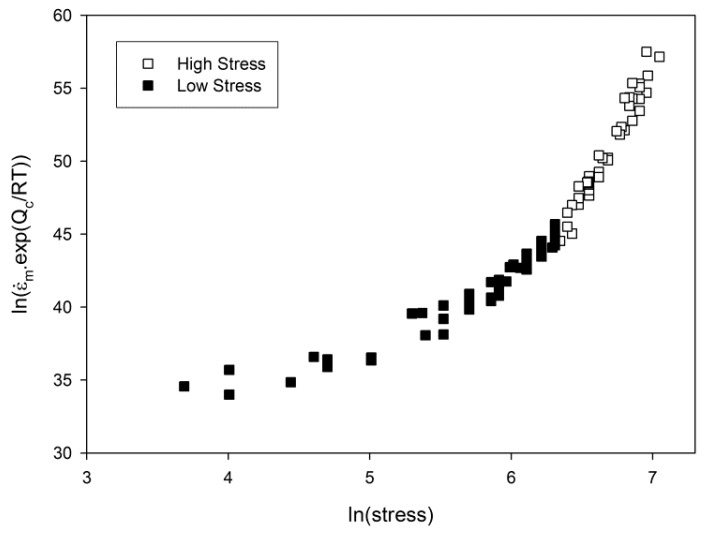
The relationship between ln(stress) and ln($\dot{\varepsilon }$_m_·exp(Q_c_/RT)) in Waspaloy. Q_c_* = 500 KJ/mol.

**Figure 5:**
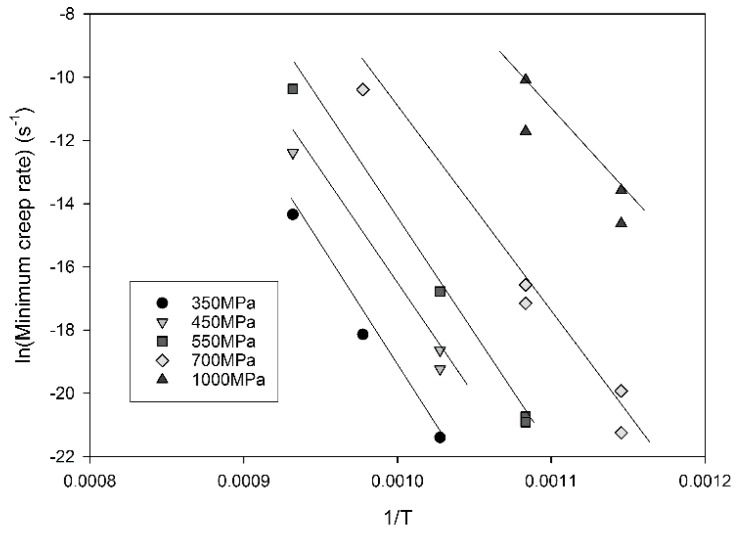
The relationship between ln$\dot{\varepsilon }$_m_ vs. 1/Temperature in Waspaloy.

The relationship between stress and minimum creep rate is displayed in Figure 3. Linear relationships can be derived allowing for the calculation of *n* values according to Equation (1) by plotting ln($\dot{\varepsilon }$_m_·exp(Q_c_/RT)) against lnσ where the *n* value is represented by the gradient of the graph (Figure 4). It is clear that a significant change in gradient occurs at higher stresses as the *n* value varies from ~5 to ~18. This region, historically known as ‘power law breakdown’, has been widely observed for a range of precipitation hardened alloys, and provides a significant challenge to designers as power law based equations fail to accommodate the rapidly changing gradient of the graph. The situation is further complicated when Equation (1) is used to derive an activation energy for the material by plotting ln$\dot{\varepsilon }$_m_ vs. 1/Temperature as shown in Figure 5. When high stresses are used for the calculation, values of Q_c_ = 350 kJ/mol are derived, whereas this increases to 700 kJ/mol for low stresses, further illustrating the problem of so-called ‘variable constants’ in Equation (1) upon which many lifing approaches are based.

**Figure 6:**
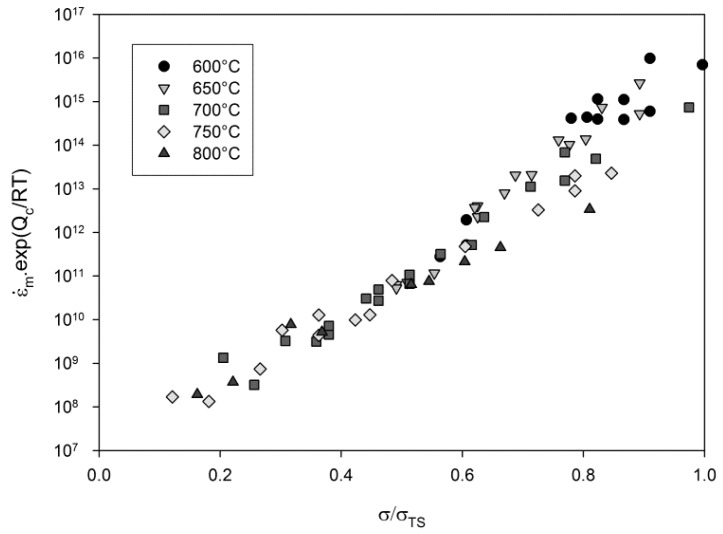
The relationship between σ/σ_TS_ and ($\dot{\varepsilon }$_m_·exp(Q_c_/RT)) in Waspaloy.

One approach to overcome these difficulties has been to normalise each temperature dataset by the UTS of the material, obtained from high-strain-rate tests at each temperature, as shown in Figure 6. It can be seen that the data produced over a range of temperatures now can be collapsed to a single curve defining a single modified activation energy. Whilst it is recognized that normalization by other temperature dependent parameters (i.e., yield stress) may also be utilized, normalization by the UTS offers a parameter which is easily measurable and encompasses all possible values of stress conveniently between 0 and 1.

## 4. Discussion

The unknown curvature of Figure 6 provides a problem for designers should data extrapolation outside of the original dataset be required and it soon becomes clear that a more robust approach should be attempted. The Wilshire equations are a natural (though not the only) choice since they are based around normalisation through the UTS which has already been shown to be effective, Equation (2).

(2) $$\left(\sigma /\sigma_{TS}\right)=\exp\left\{-k_{2}{[{\dot{\varepsilon }}_{m}\exp\left(Q_{c}^{*}/RT\right)]}^{v}\right\}$$

**Figure 7:**
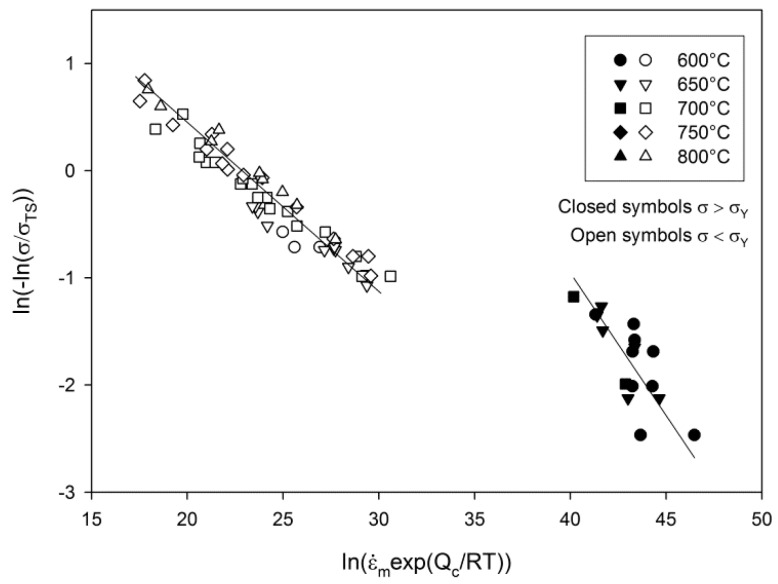
Deriving the constants for the Wilshire equations.

**Figure 8:**
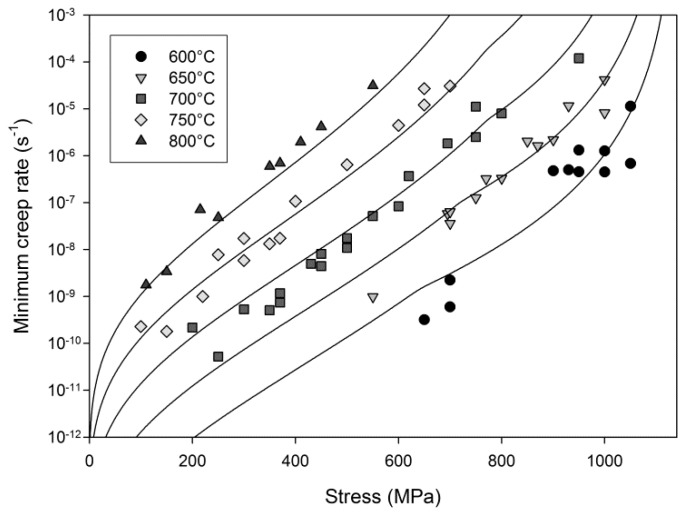
Predictions of minimum creep rate as a function of stress.

In attempting to derive the Wilshire equation fits for the Waspaloy tests described here, a break in the data provides a more accurate fit in conjunction with the parameters derived in Figure 7. A linear relationship for ‘high stress’ data can be observed for which *v* = −0.24, *k*_2_ = 434 and Q_c_* = 400 kJ·mol^−1^, with a second line accurately fitting lower stress data where *v* = 72, *k*_2_ = −0.14 and Q_c_* = 340 kJ·mol^−1^. The use of differing values of Q_c_* reduces scatter in the data and allows for more accurate fits, and should be compared with the increased scatter which occurs when using a single value in Figure 6. Clearly from previous experience and numerous examples in the open literature [11,16,18,19,20,24,25], the transition point between the two lines would be expected to approximate the onset of yielding in the material at each individual temperature, and simple calculations show that to be the case in the current material. (Values for the yield stress and 0.2% proof stress are provided in Table 2, values for 650 °C are not included due to a strain monitoring issue during the test.) Furthermore, since the value of the apparent activation energy, Q_c_*, is derived from each individual linear segment, a change in activation energy was observed above (420 kJ/mol) and below (340 kJ/mol) the yield stress, as has been found for previous materials [24,27]. The fits for stress vs. minimum strain rate based on the Wilshire equations can be seen in Figure 8.

**Figure 9:**
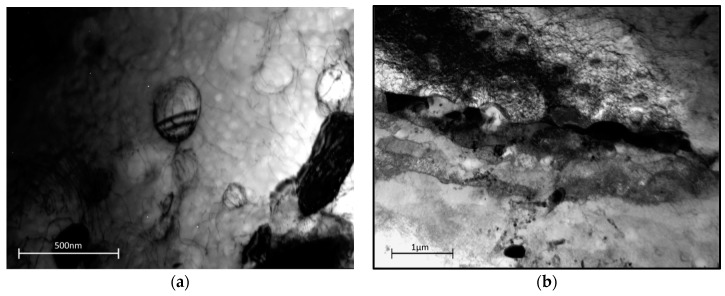
Dislocation structure in as-received Waspaloy material (**a**) within the grains (**b**) in the grain boundary zone.

To investigate whether there is an underlying physical mechanism change driving the variation in activation energy, a series of creep tests was undertaken at graded levels of stress both above and below the yield stress at a temperature of 700 °C. Figure 9a,b shows the dislocation structure of the as-received material under transmission electron microscope (TEM) imaging. In Figure 9a, a bimodal distribution of γ′ precipitates is visible with the secondary precipitates approximately 200 nm in diameter and the tertiary of the order of 50 nm diameter. Dislocations are visible in the γ matrix at moderate density but they generally avoid passing through the larger secondary precipitates, wrapping around in loose networks. The microstructure is consistent with the thermo-mechanical processing route. Figure 9b shows an area straddling a grain boundary. In an area 1–2 µm around the boundaries, there is evidence of additional strain which has undergone significant recovery to produce a series of sub-grains separated by low angle boundaries. Carbides are visible on the grain boundary.

**Figure 10:**
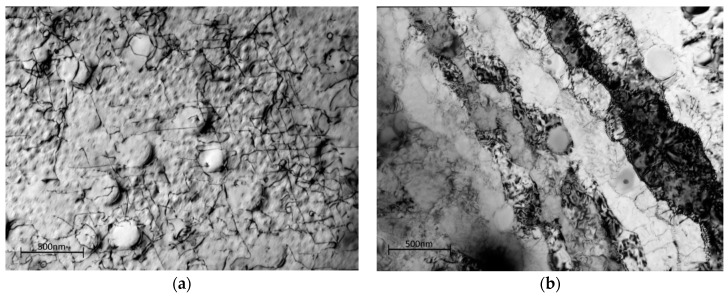
Dislocation structure in Waspaloy subjected to loading to 0.2% proof stress in the (**a**) grains; (**b**) grain boundary zones (bright field images).

In order to identify the separate effects of creep and plasticity, a monotonic tensile test was performed where the test was halted at the approximate 0.2% proof stress (740 MPa) of Waspaloy at 700 °C, unloaded and removed from the test machine. The dislocation structure mid-grain and at the boundaries can be seen in Figure 10a,b. Mid-grain there is an increase in the dislocation density, as appropriate sources are activated at the yield point. The dislocations are looping around the secondary precipitates, but for the most part, are cutting through the tertiary γ′ as single dislocations with the exception of some of the larger precipitates: an example of this is evident in the top right of Figure 10a. There is no evidence of stacking faults in any part of the microstructure and it is interesting to note that the dislocation does not appear to be moving in pairs. Figure 10b shows the grain boundary area of the yielded sample. The low angle sub-grains remain as in the virgin material.

Creep tests were performed at 500, 600, 700 and 800 MPa in which the material was loaded and allowed to creep until the minimum creep rate was reached; Table 3 marks the end of the primary phase. As this material does not show sustained steady state creep, as shown in Figure 2, this also marks the onset of tertiary creep, where damage begins to accumulate leading to an acceleration in creep rate and final failure.

Following the attainment of the minimum creep rate, specimens were air cooled immediately under a reduced load to preserve the dislocation structure. For the creep test performed at 500 MPa, the test was interrupted at 21 h (0.25% creep strain), the time associated with the minimum strain rate from a similar test to rupture. At this point, the dislocation structures should be fully evolved but the effects of tertiary behaviour avoided.

**Figure 11:**
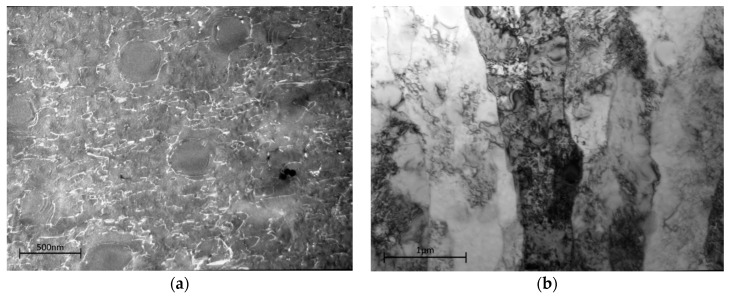
Dislocation structure in Waspaloy subjected to creep loading at 500 MPa in the (**a**) weak beam image of the interior of a grain; (**b**) bright field image of the grain boundary zone.

Figure 11a,b shows the TEM images for the dislocation structure at this creep condition. In the grain centres, the dislocation density (bright lines in this weak-beam image) is relatively low and those present are hindered by the γ′. There are frequent instances of Orowan looping around not only all the secondary γ′, but also the larger tertiary γ′ and occasional instances of stacking faults in the tertiary γ′.

The grain boundary zones at this condition retain the sub-grain structure of the virgin material, but also show similar creep dislocation structures within the sub-grains. It is unclear the extent to which evolution of the sub-grain structure in this area is contributing significantly to the creep strain. As the overall creep strain remains low at about 1%, it would require a detailed study to establish whether any enhanced grain boundary activity was in addition to that required to accommodate different strains in adjacent grains.

**Figure 12:**
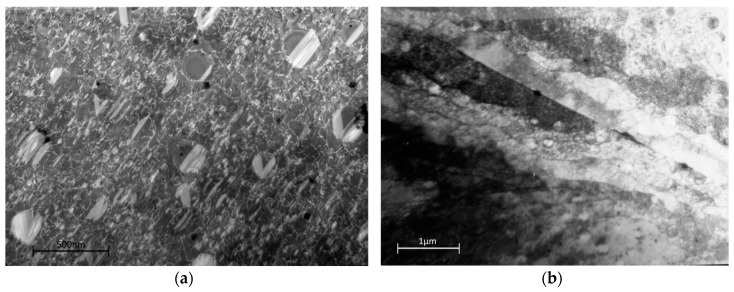
Dislocation structure in Waspaloy subjected to creep loading at 600 MPa (below the yield stress) in the (**a**) grains (**b**) grain boundary zones.

At 600 MPa and 700 MPa, the specimens were crept to the minimum strain rate in 22 and 3 h (1.02% and 2% creep strain) respectively. In comparison to the 500 MPa condition, dislocation density has increased with stress. Figure 12a,b shows the dislocation structures formed during the 600 MPa test, below the 0.2% proof stress (740 MPa). Stacking faults have become much more widely distributed in both the secondary and tertiary γ′ as the precipitates are cut by superlattice partial dislocations, and Orowan looping is consequently reduced, as shown in Figure 12a. In some grains, slip is occurring on more than one slip system as evidenced by the stacking faults on a second plane. The dislocation spacing in between the secondary precipitates, although increased over that at the 500 MPa interrupted test, remains below the tertiary precipitate spacing, indicating that the principal barrier to slip is still the tertiary precipitates.

Grain boundary zones are populated by the low angle sub-grains with dislocation banding still present at the sub-grain boundaries, as visible in Figure 12b. The overall dislocation structure is now seemingly a combination of dislocation recovery and strain hardening processes, showing considerable changes when compared to the 500 MPa test specimen.

The specimens crept at 700 MPa and 800 MPa reached the minimum strain rate in very similar times of 3 and 2 h (2% and 4.4% creep strain) respectively. In contrast, the stress conditions below the yield point at 500 and 600 MPa required 21 and 22 h respectively.

**Figure 13:**
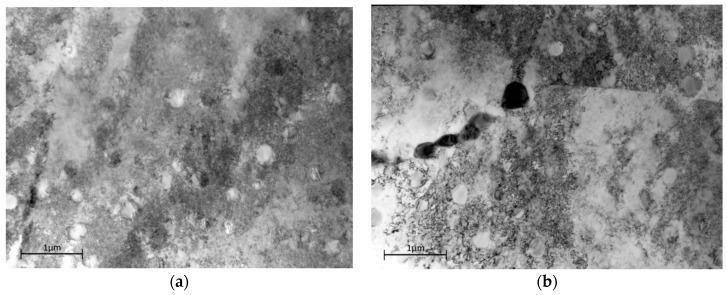
Dislocation structure in Waspaloy subjected to creep loading at 800 MPa (above the yield stress) in the (**a**) grains (**b**) grain boundary zones.

Figure 13a,b highlights the dislocation structure present above the yield stress at 800 MPa, crept until the minimum strain rate was reached. In the bulk of the material, the dislocation density is so great that individual dislocations become increasingly difficult to resolve. A second creep test was performed to only 1% strain (attained within 20 min) to help resolve the dislocation behaviour but was substantially similar.

Dislocations are extremely dense but mostly confined to the γ and the tertiary γ′ with occasional stacking faults in the secondary γ′. These larger precipitates continue to resist cutting by lattice dislocations as observed in the tensile test to yield, as shown in Figure 10a.

The best fit of the creep rupture data to the Wilshire equations for Waspaloy (and many other alloys) is obtained by defining two separate linear relationships describing the high and low stress behaviour. The stress transition point giving the best fit falls naturally at the approximate yield stress of the material, 735 MPa. This implies a change in creep behaviour associated with the yield stress as evidenced by the change in activation energy. The dislocation structures after creep above and below the original yield stress do indeed show a distinct difference. Below the yield stress, densities are relatively low, increasing slightly with stress, and the time to minimum creep strain is of the order of 20 h. The pre-existing subgrain structure in the vicinity of the grain boundaries is retained. Above the yield point, the dislocation density increases to the point where the dislocation spacing is closer than that of the precipitates and the sub-grain structure around the grain boundaries is obliterated by the increased density throughout. Minimum creep rates are achieved after about 3 h. The secondary precipitates alone remain clear of all but the occasional stacking fault.

These observations can be rationalised as follows. During creep below the yield stress, the dislocation densities remain very low and the main resistance to dislocation motion is the interaction with the tertiary precipitates. The secondary precipitates alone are too widely spaced to offer much additional hardening. Indeed, at 500 MPa the stress appears to be too low to allow cutting of the tertiary precipitates, even by partial dislocations trailing low energy superlattice stacking faults, let alone the higher energy Anti Phase Boundary (APB) faults. Dislocation motion is therefore limited by the climb of these dislocations over and around all but the smallest tertiary precipitates. Densities increase with stress but nevertheless remain low as sources of new dislocations cannot readily be activated by stress below yield. As the stress rises to 600 MPa, the precipitates are increasingly below the size threshold for cutting by superlattice partial dislocations and the microstructure shows numerous stacking faults in the precipitates as during creep there is sufficient time to allow the reordering necessary for this to happen [32].

Above yield, the stress is sufficient for the dislocations to cut through the tertiary precipitates as shown in the tensile test to yield, as shown in Figure 10. This reveals that the process of yield is associated with the ability of dislocations to cut the tertiary precipitates which provide the principal increment of strength in a bimodal distribution of ordered precipitates [33]. The secondary precipitates are too large to cut and dislocations loop around them leaving them dislocation free. At the high strain rate in a tensile test, the activated movement of partial dislocations is not possible, and the dislocations cut through the tertiary precipitates as single dislocations producing APB faults which are rectified by subsequent dislocations, i.e., ‘weak coupling’ [34]. In the creep tests above yield, the rapid multiplication of dislocations, triggered by the freedom of the dislocations to glide through the tertiary precipitate structure, leads to a greatly increased dislocation density. This, in turn, leads to the increase in flow strength associated with forest hardening due to the exceptionally high dislocation densities between the tertiary precipitates adding to the precipitate hardening of the virgin material. Note that the stress is still insufficient for the dislocations to pass through the secondary precipitates leaving these relatively dislocation free. At these higher stresses, the minimum strain rate will occur when the dislocation multiplication rate is balanced by the recovery rate. Initially, the flow rate increases due to higher mobile dislocation density, but eventually the density becomes so high that dislocations become locked in tangles lowering the creep rate. The closer spacing of the dislocations facilitates recovery until equilibrium is reached.

Based on this interpretation, the critical activation event below the original yield stress is associated with the climb of the dislocations around the tertiary γ′ precipitates: above the original yield point with the climb and recovery of dislocation tangles. The extent of dislocation interaction during the high stress regime of any material in creep is reflected in the rate of strain hardening of a material observed during monotonic tensile tests. There is a qualitative relationship between the apparent activation energy and the amount of strain hardening a material undergoes after yield, as evidenced by three model materials; 316 stainless steel has a very high rate of strain hardening and shows Q_c_* values of 250/150 kJ/mol [24]; Waspaloy has a medium rate of strain hardening with Q_c_* values of 400/340 kJ/mol; and Titanium 6-4 has a very low rate of strain hardening with negligible activation energy change, having Q_c_* values of 250/250 kJ/mol [26]. This supports the proposal that activation energy change in creep is a direct result of the onset of strain hardening: an alloy with no potential for strain hardening would not be expected to show a change in activation energy.

## 5. Conclusions

The following conclusions can be drawn from this programme of work:

- Creep deformation in Waspaloy is dominated by diffusion controlled dislocation movement.
- The best fit of the creep rupture data to the Wilshire equations for Waspaloy is obtained by two separate linear relationships separated at the yield point.
- Below yield creep takes place through the movement of dislocations controlled by diffusive climb around precipitates, whereas above yield dislocation movement is limited by forest hardening.
- The change in apparent activation energy, Q_c_*, is directly related to the amount of strain hardening in an alloy brought about by high dislocation densities generated at stresses above yield.
- The change in activation energy, and the concomitant breaks in the Wilshire equations, are brought about by a change the mechanism controlling dislocation movement, not a complete change of mechanism.

## Figures and Tables

**Table 1:** Chemical Composition of Waspaloy (in wt %) [28].

Cr	Co	Mo	Al	Ti	C	B	Ni
19.5	13.5	4.3	1.3	3	0.08	0.006	Bal

**Table 2:** Yield stress values for Waspaloy.

Temperature (°C)	Yield Stress (MPa)	0.2% Proof Stress (MPa)
550	745	912
600	750	920
700	735	874
800	520	675

**Table 3:** Time to minimum creep rate and associated strain for the range of stress conditions.

Applied Stress (MPa)	Time to Minimum Creep Rate (h)	Strain at Minimum Creep Rate (%)
500	21	0.25
600	22	1.02
700	3	2
800	2	4.4
